# Short-term outcomes of on- vs off-pump coronary artery bypass grafting in patients with left ventricular dysfunction: a systematic review and meta-analysis

**DOI:** 10.1186/s13019-020-01115-0

**Published:** 2020-05-11

**Authors:** Zhiyuan Guan, Xiaoqing Guan, Kaiyun Gu, Xuanqi Lin, Jin Lin, Wenjun Zhou, Ming Xu, Fen Wan, Zhe Zhang, Chunli Song

**Affiliations:** 1 Department of Cardiology surgery, Peking University Third Hospital, 49 North Garden Rd., Haidian District, Beijing, 100191 China; 2 Peking University, Beijing, 100871 China; 3 Beijing University of Technology, Beijing, 100124 China; 4 Department of Cardiology, Peking University Third Hospital, NHC Key Laboratory of Cardiovascular Molecular Biology and Regulatory Peptides, Beijing, 100191 China; 5 Shanghai East Hospital, Tongji University, 150 Jimo Rd., Pudong District, Shanghai, 100124 China; 6 Department of Orthopedics, Peking University Third Hospital, 49 North Garden Rd., Haidian District, Beijing, 100191 China

**Keywords:** On-pump, Off-pump, Left ventricular dysfunction, Coronary artery bypass grafting

## Abstract

**Objectives:**

Does the manipulation of the off-pump CABG (OPCAB) in patient with depressed left ventricular function is better than on-pump CABG (ONCAB) approach in in-hospital mortality and morbidities? Here we undertook a meta-analysis of the best evidence available on the comparison of primary and second clinical outcomes of the off-pump and on-pump CABG.

**Design:**

Systematic literature reviewer and meta-analysis.

**Data sources:**

PubMed, EMBASE, Web of science and Cochrane Center Registry of Controlled Trials were searched the studies which comparing the use of the off-pump CABG(OPCAB) and on-pump CABG (ONCAB) for patients with LVD during January 1990.1 to January 2018.

**Eligibility criteria:**

All observation studies and randomized controlled trials comparing on-pump and off-pump as main technique for multi-vessel coronary artery disease (defined as severe stenosis (>70%) in at least 2 major diseased coronary arteries) with left ventricular dysfunction(defined as ejection fraction (EF) 40% or less) were included.

**Data extraction and synthesis:**

Authors will screen and select the studies extract the following data, first author, year of publication, trial characters, study design, inclusion and exclusion criteria, graft type, clinical outcome, assess the risk of bias and heterogeneity. Study-specific estimates will pool through the modification of the Newcastle-Ottawa scale for the quality of study and while leave-one-out analysis will be used to detect the impact of individual studies on the robustness of outcomes.

**Results:**

Among the 987 screened articles, a total of 16 studies (32,354 patients) were included. A significant relationship between patient risk profile and benefits from OPCAB was found in terms of the 30-day mortality (odds ratio [OR], 0.84; 95% confidence interval [CI], 0.73–0.97; *P* = 0.02), stroke (OR, 0.69; 95% CI, 0.55–0.86; *P* = 0.00)**,** myocardial infarction (MI) (OR, 0.71; 95% CI, 0.53–0.96; *P* = 0.02), renal failure (OR, 0.71; 95% CI, 0.55–0.93; *P* = 0.01), pulmonary complication (OR, 0.68; 95% CI, 0.52–0.90; *P* = 0.01), infection (OR, 0.67; 95% CI, 0.49–0.91; *P* = 0.00),postoperative transfusion (OR, 0.25; 95% CI, 0.08–0.84; *P* = 0.02) and reoperation for bleeding (OR, 0.56; 95% CI, 0.41–0.75; *P* = 0.00). There was no significant difference in atrial fibrillation (AF) (OR, 0.96;95%; CI, 0.78–1.41; *P* = 0.56) and neurological dysfunction (OR, 0.88; 95% CI, 0.49–1.57; *P* = 0.65).

**Conclusions:**

Compared with the on-pump CABG with LVD, using the off-pump CABG is a better choice for patients with lower mortality, stroke, MI, RF, pulmonary complication, infection, postoperative transfusion and reoperation for bleeding. Further randomized studies are warranted to corroborate these observational data.

## Introduction

The impresses left ventricular function is important risk factors to effect the clinical outcome of coronary artery bypass surgery. Several meta-analysis has been performed that investigated the short-term and long-term clinical prognosis of on-pump versus off-pump CABG [1, 2]. Topkara et.al found that in-hospital mortality and morbidities were significantly higher in patients underwent CABG with depressed LV function than normal LV function [3]. For patient with lower left ventricular function, comparing medical therapy with CABG for patients with symptomatic coronary artery disease and ejection fraction (EF) as low as 30% have shown a long-term survival benefit for those receiving CABG [4].

The CABG focus on long term benefits compared with medical treatment of coronary artery disease in patients with lower left ventricular function [5] and up to 15% of patients present with severely depressed left ventricular function [6] .Due to the improved technique and LVAD/ECMO led to progressively improved CABG clinical outcome in recent years. on the other hand, it has been suggested that off-pump CABG may be beneficial in patients with severely depressed LV function by avoiding prolonged ischemic times. In the 2011, Jarrel OA et.al [7] has been aggregated meta-analysis which has focused on the comparison of clinical results of the CABG, especially in patients with LVD show that off-pump CABG may be associated with lower incidence of early mortality in patient with LVD. Therefore, the advantages of off-pump compared with conventional on-pump CABG in patients with LVD remain a source of controversy. On this background, the aim of this systematic review was to synthesize the results from all studies reporting the short-term clinical outcome that investigated on- versus off-pump CABG in patients with LVD.

## Methods

This systematic review and meta-analysis follow the preferred reporting items for systematic reviews and meta-analysis statement.

### Search strategy and definition

A medical librarian developed searches to identify studies that compared the clinical outcome between on-pump and off-pump CABG. PubMed, EMBASE, Web of science and Cochrane Center Registry of Controlled Trials were searched during January 1990.1 to January 2018. Searches used subject headings and keywords for the following terms: ‘coronary artery bypass, off-pump, on-pump, left ventricular dysfunction, cardiopulmonary bypass, CABG.’(Supplement 1 search strategy).

To be eligible for inclusion in our meta-analysis, trials had to conform to the following criteria: the observation studies comparing on-pump and off-pump as main technique for multivessel coronary artery disease (defined as severe stenosis (>70%) in at least 2 major diseased coronary arteries) with left ventricular dysfunction(defined as ejection fraction 40% or less). Animal studies, review papers were excluded. Studies that did not have any of the desired outcome measures or participants who were treated by other modalities such as percutaneous coronary intervention and emergency or salvage conditions were excluded. Incomplete data were excluded. Studies that included interventions other than off-pump versus on-pump CABG were excluded.

### Data extractions and quality assessment

**Fig. 1:**
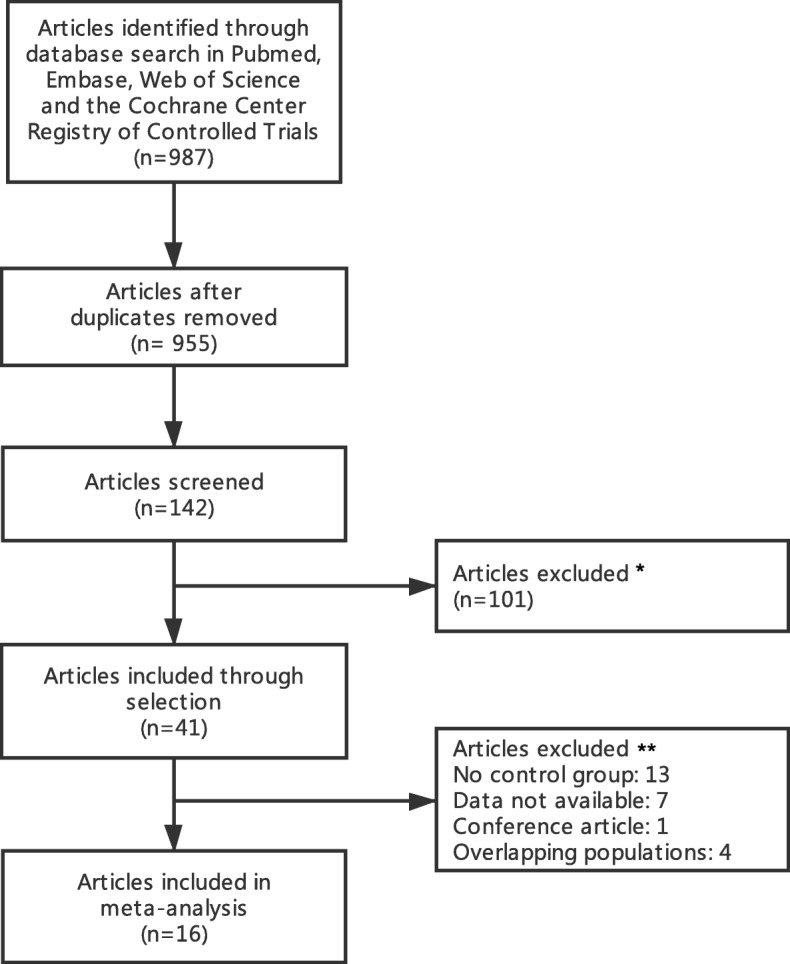
Preferred reporting items for systematic reviews and meta-analysis flow diagram

Three reviewers (Guan; Gu; Lin) independently extracted the following data from each study, first author, year of publication, trial characteristics, study design, inclusion and exclusion criteria, graft type, clinical outcome (Fig. 1). The following variables were included: study demographics (sample size, publication year, design, and country), patient demographics and comorbidities (age, sex, diabetes, ejection fraction, chronic obstructive pulmonary disease). In the first screening phase, we have excluded 101 papers due to they were irrelevant. The modification of the Newcastle-Ottawa scale is carried out in our meta-analysis with a quality assessment score. The modified Newcastle-Ottawa scale checklist has been summarized in Table 1**,** and we also define the studies scores higher than 6 as the high-quality study. The quality of all studies has been evaluated by two independent researchers (Zhou; Lin).

**Table 1:** Quality assessment of included studies using Newcastle-Ottawa scale

First author	Year	Selcetion				Comparability		Outcome			Total
		S1	S2	S3	S4	C1	C2	O1	O2	O3	
Arom, K.V.	2000	1	1	0	1	1	0	1	1	1	7
Yokoyama, T.	2000	1	1	0	1	1	0	1	1	1	7
Shennib, H.	2002	1	1	0	1	1	0	1	1	1	7
Al-Ruzzeh, S.	2003	1	1	0	1	1	0	1	1	1	7
Ascione, R.	2003	1	1	0	1	1	1	1	1	1	8
Goldstein, D.J.	2003	1	1	0	1	1	1	1	1	1	7
Darwazah, A.K.	2006	1	1	0	1	1	1	1	1	1	8
Sharoni, E.	2006	1	1	0	1	1	0	1	1	1	7
Filsoufi, F.	2007	1	1	1	1	1	0	1	1	1	8
Youn, Y.N.	2007	1	1	0	1	1	0	1	1	1	7
Qiu, Z.B.	2008	1	1	0	1	1	0	1	1	1	8
Attaran, S.	2010	1	1	0	1	1	0	1	1	1	7
Caputti, G.M.	2011	1	1	0	1	1	0	1	1	1	7
Emmert, M.Y.,	2012	1	1	0	1	1	0	1	1	1	7
Keeling, W.B.	2013	1	1	0	1	1	0	1	1	1	7
Ueki, C.	2016	1	1	0	1	1	0	1	1	1	7

S1: Representativeness of the exposed cohort; S2: Selection of the non-exposed cohort, S3: Ascertainment of exposure, S4: Demonstration that outcome of interest was not present at start of study; C1&2: Comparability of cohorts on the basis of the design or analysis; O1: Assessment of outcome, O2: Was follow-up long enough for outcomes to occur, O3: Adequacy of follow-up of cohorts

### Outcomes

The primary clinical endpoint was 30-days mortality. The secondly clinical endpoint was stroke, myocardial infarction and renal failure, atrial fibrillation, renal failure, pulmonary complications, postoperative transfusion, neurological dysfunction and infection. Pulmonary complications were include respiratory failure (pulmonary insufficiency requiring intubation and ventilation for a period of 72 h or more at any time during the postoperative stay) and postoperative pneumonia (positive sputum cultures with subsequent antibiotic treatment, or an infiltrate on postoperative chest x-ray diagnosed as pneumonia or pneumonitis). The period of secondly clinical outcome were defined as 30 days after surgery.

### Statistical analysis

The relationship between on-pump and off-pump CABG and clinical outcome was compared directly by pooling data from the included studies using “meta” and “metaphor” packages in R (version 3.5.3, R Project; R Foundation for Statistical Computing, Vienna, Austria) [8]. We pooled the clinical outcome using OR with 95% CI. OR were used as the common measure for dichotomous data follow by the previous study [7] and Cochrane Handbook for Systematic Reviews of Intervention [9]. The random-effects model because variation among studies due to patients undergoing operations in different centers have varying risk profiles and selection criteria for each surgical technique. We evaluated the heterogeneity by focusing on patients with LVD and a quality score greater than 7 and Heterogeneity was reported as low (I^2^ = 0–25%), moderate (I^2^ = 26–50%), high (I^2^ > 50%), consistent with guidelines. Publication bias was assessed visually by funnel plot and quantitatively by the Egger test [10]. We calculated pooled ORs using the Mantel-Haenszel method. A leave-one-out analysis was performed to examine the impact of individual studies on the robustness of the primary and secondary outcomes. Statistical significance was assumed for *P* < 0.05.

## Results

Among the 987 screened articles, article excluded due to screened the title(29 studies),abstract(60 studies),key word(12 studies) at first time and full text(25 studies) at second time. a total of 16 studies(32,354 patients; 24,295 case of on-pump CABG and 8269 cases of off-pump CABG)were included (Table 2).

**Table 2:** Study characteristics and patient demographics of included studies

Author	Year	Arm	Total patients	Age, mean (SD)	Gender, female	Smoker	COPD	Hypertension	Diabetes	Dyslipidemia	Renal dysfunction	MI	CVA	TDV	LVEF
Arom, K.V.	2000	off-pump	45	70.20 (11.80)	16	10	6	30	15	NR	NR	NR	4	NR	24.80 ± 5%
		on-pump	132	66 (11.60)	27	24	17	71	45	NR	NR	NR	11	NR	26.40 ± 4%
Yokoyama, T.	2000	off-pump	242	67	NR	NR	34	NR	83	NR	27	NR	NR	NR	≤25.00%
		on-pump	483	68	NR	NR	44	NR	140	NR	46	NR	NR	NR	≤25.00%
Shennib, H.	2002	off-pump	31	64.6 0(9)	4	9	1	13	13	17	3	25	2	NR	28.80 ± 6.10%
		on-pump	46	64.50 (9.90)	7	20	7	24	13	20	4	45	4	NR	28.40 ± 5.80%
Al-Ruzzeh, S.	2003	off-pump	106	NR	24	78	7	65	32	73	2	56	15	NR	21.60 ± 1.80%
		on-pump	199	NR	67	153	19	97	61	106	13	127	24		21.80 ± 1.20%
Ascione, R.	2003	off-pump	74	66	10	61	NR	51	23	54	NR	61	7	50	≤30.00%
		on-pump	176	65	14	132	NR	94	41	130	NR	139	27	141	≤30.00%
Darwazah, A.K.	2006	off-pump	66	56.10 (10.80)	14	43	15	34	30	29	10	44	NR	NR	27.50 ± 5.50%
		on-pump	84	58.70 (9.40)	25	44	8	38	44	36	8	42	NR	NR	30.10 ± 4.2%
Sharoni, E.	2006	off-pump	144	63 (10.60)	40	55	58	109	67	NR	19	106	NR	107	28 ± 7%
		on-pump	209	61.90 (10.90)	36	73	48	143	80	NR	26	162	NR	155	28 ± 6%
Filsoufi, F.	2007	off-pump	71	69 (11)	27	NR	8	54	36	NR	9	47	9	48	≤30.00%
		on-pump	424	65 (11)	117	NR	40	327	193	NR	33	339	32	329	≤30.00%
Youn, Y.N.	2007	off-pump	100	62.90 (8.80)	27	50	3	NR	56	46	14	59	12	85	≤35.00%
		on-pump	53	62.00 (9.20)	15	20	3	NR	26	22	7	27	4	46	≤35.00%
Qiu, Z.B.	2008	off-pump	84	NR	20	62	16	54	27	59	5	45	8	NR	30.91 ± 1.24%
		on-pump	102	NR	37	79	23	53	35	56	10	65	6	NR	30.62 ± 1.58%
Attaran, S.	2010	off-pump	406	67	60	79	172	251	132	375	51	69	45	336	≤30.00%
		on-pump	528	66.10	70	107	222	286	143	458	51	75	54	475	≤30.00%
Caputti, G.M.	2011	off-pump	105	71 (3)	27	26	14	70	31	40	12	42	4	NR	≤20.00%
		on-pump	112	67 (2)	23	29	13	59	38	48	10	40	8	NR	≤20.00%
Emmert, M.Y.,	2012	off-pump	256	64 (10)	49	157	13	149	91	167	11	176	6	206	≤35.00%
		on-pump	222	63 (9)	34	133	24	112	50	159	10	203	0	54	≤35.00%
Keeling, W.B.	2013	off-pump	5158	65(11.10)	1161	NR	NR	4393	2560	NR	277	3419	882	NR	23% (20–25%)
		on-pump	20,509	64	4138	NR	NR	17,245	10,716	NR	923	13,644	3287	NR	23% (20–25%)
Ueki, C.	2016	off-pump	1053	67.40(10.10)	150	701	NR	758	633	571	128	615	182	840	27.20 ± 7.90%
		on-pump	1134	65.70(10.20)	156	742	NR	835	731	669	160	693	150	955	26.60 ± 10.40%

*COPD* chronic obstructive pulmonary disease, *CVA* Cerebrovascular accident, *TDV* Three diseased vessel, *NR* not reported

Six of the studies were multicenter. Five studies formed the USA, three from UK and two from Israel, and one each from Canada, Korea, Brazil, China, Switzerland and Japan. All observational studies included were of high quality and low risk of bias. The number of patients in the individual studies ranged from 26 to 20,509 patients in the on-pump CABG group and from 31 to 5158 in the off-pump CABG group. The overall mean age ranged from 65.62 years in the on-pump CABG group and 64.23 in the off-pump CABG group. In the off pump group, the overall percentage of female varied from 12.9–38%, whilst in the on pump group the percentage of female ranged from 8 to 36.3%. All patients had low-normal ejection fraction (range from ≤20% to ≤35%).

For short-term outcomes, mortality was reported in 15 studies (31,668 patients) [11–21] and pulmonary complication in 9 studies (3987patients) [11, 12, 15, 16, 19, 20, 22, 23], renal failure in 15 studies (31,801 patients) [5, 11–18, 20–22, 24], infection in 8 studies (5037 studies) [5, 11, 12, 14, 15, 20, 25], AF in 12 studies (30,789 patients) [12, 14–20, 22–25], postoperative transfusion in 4 studies (2565 patients) [20, 21, 24, 25], reoperation for bleeding in 11studies(5418 patients) [5, 11, 13, 14, 16, 20, 21, 23, 24], MI in 13 studies (31,686 patients) [5, 11–20, 22, 23, 25] and neurological dysfunction in 7 studies (1536 patients) [12, 14–20, 22, 23, 25].

### Primary outcomes

**Fig. 2:**
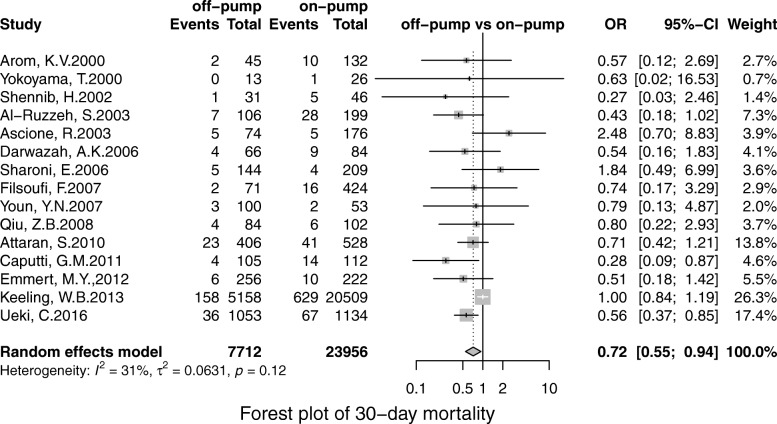
Forest plot for 30-day mortality

30-day mortality was 3.34% in off-pump group versus 3.53% in on-pump group (OR, 0.84; 95%CI, 0.73–0.97; *P* = 0.02) and Leave-one-out analysis supported the robustness of this finding(Figure 2). Funnel plot showed no publication bias (Egger test intercept was − 1.53-0.12, *P* = 0.12,Supplementary Figure 1a). However, when excluding the study of Ueki, C. et.al, the off-pump was no longer associated with a significantly lower risk of 30-day mortality. (Supplementary Figure 1b).

### Secondary outcomes

**Fig. 3:**
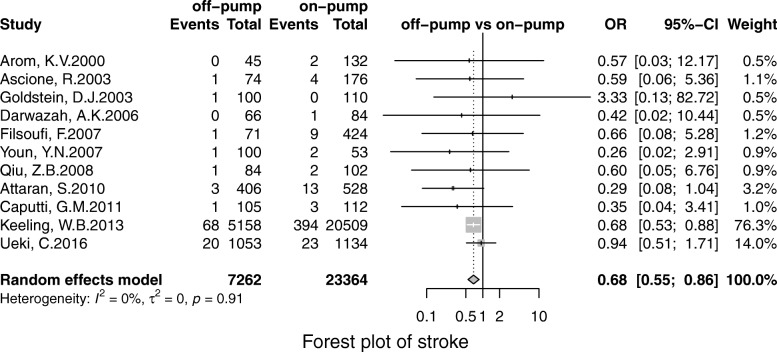
Forest plot for shock

**Fig. 4:**
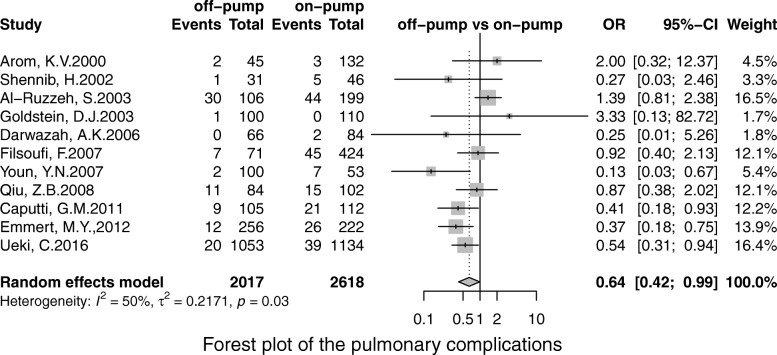
Forest plot for the pulmonary complication

**Fig. 5:**
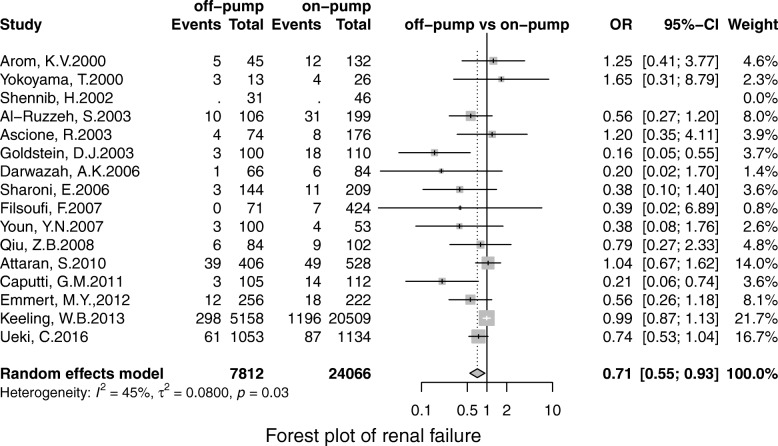
Forest plot for renal failure

**Fig. 6:**
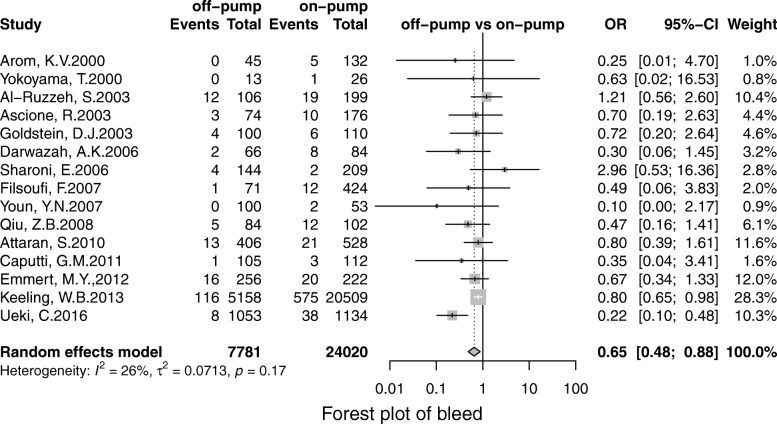
Forest plot for infection

**Fig. 7:**
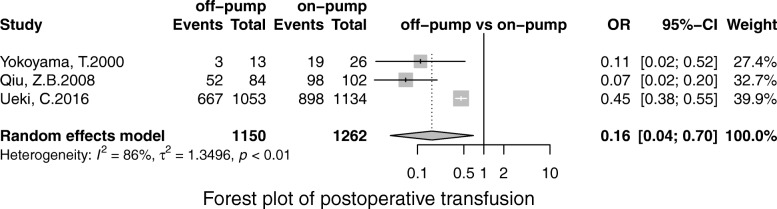
Forest plot for AF

**Fig. 8:**
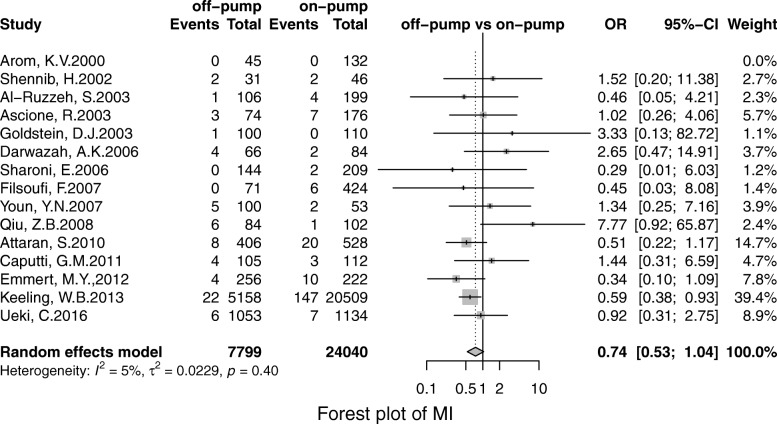
Forest plot for MI

**Fig. 9:**
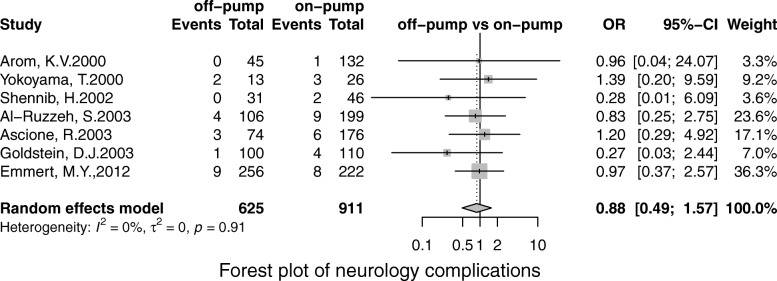
Forest plot for neurology complications

**Fig. 10:**
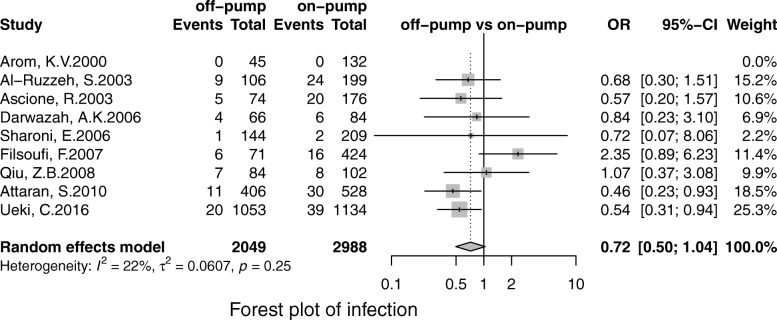
Forest plot for reoperation for bleeding

**Fig. 11:**
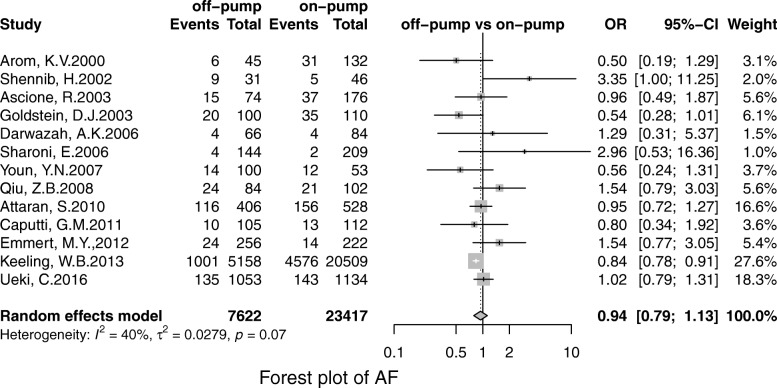
Forest plot for postoperative transfusion

Off-pump was associated with less stroke (OR, 0.69; 95% CI, 0.55–0.86; *P* = 0.00), MI (OR, 0.71;95% CI, 0.53–0.96; *P* = 0.02), renal failure (OR, 0.71; 95% CI, 0.55–0.93; *P* = 0.01), the pulmonary complication (OR, 0.68; 95% CI, 0.52–0.90; *P* = 0.01), infection (OR, 0.67; 95% CI, 0.49–0.91;*P* = 0.00), postoperative transfusion (OR, 0.25; 95% CI, 0.08–0.84; *P* = 0.02), reoperation for bleeding(OR, 0.56; 95% CI, 0.41–0.75; *P* = 0.00) respectively. However, there was no significant difference in terms of AF (OR, 0.95; 95% CI, 0.78–1.41; *P* = 0.56) and neurological dysfunction (OR, 0.84; 95% CI, 0.49–1.57; *P* = 0.65) (Figs. 3, 4, 5, 6, 7, 8, 9, 10, 11).

## Discussions

This study showed that off-pump CABG can be performed with better operative mortality than on-pump CABG among patients with severe LVD in our meta-analysis of contemporary observational clinical studies involving a large cohort of patients. OPCAB were also demonstrated that the rate of stroke, myocardial infarction, renal failure, pulmonary complication, infection, postoperative transfusion and reoperation for bleeding have better advantage than ONCAB.

The results of the present study are consistent with large individual studies included in the current meta-analysis. Kunadian et.al found that CABG can be performed with acceptable operative mortality and 5-year actuarial survival in patients with severe LV dysfunction in the meta-analysis [26]. The Japan Adult Cardiovascular Surgery Database registry of 918 patients with low EF (less than 0.30) reported an operative mortality of 3.30% with off-pump CABG than on-pump CABG (6.10%) [20]. Keeling et al. in the series of 25,667 patients demonstrated that off-pump CABG compared with on-pump CABG was associated with superior predicted mortality risk (2.30% vs 2.10%, *P* = 0.0001) and Major adverse cardiac events (MACE) (4.40% vs 5.30%, *P* = 0.01) including stroke, MI and atrial fibrillation [17]. Likewise, in a series of 217 patients with EF ≤ 20%, Capptti et al. demonstrated the operative mortality of 12.50% in the cardiopulmonary bypass group and 3.80% in the off-pump group [27].

The less release of inflammatory mediators, cardioplegia, hypothermia, well blood supply for the sub-endocardium and minimally invasive procedure without cross-clamping, cardiologic arrest and improve flow in IMA grafts make the off-pump CABG an almost-ideal technique for surgery [28–32]. Compared with off-pump CABG, on-pump CABG also has the additional advantage of complete revascularization, hemodynamic deterioration and repeated surgical interventions [30]. Off-pump CABG was also associated with a significantly lower incidence of renal failure, stroke, myocardial infarction, pulmonary complications, postoperative transfusion, infection in this high-risk cohort. A recent large-scale clinical trial study by Garg demonstrated that off-pump reduced the risk of acute postoperative kidney than on-pump CABG, but no evidence shows better-preserved kidney function at 1 year follow Avoidance of transfusion and eliminate extracorporeal circulation is thought to be the main reasons for the lower incidence of renal failure [28, 33, 34]. Numerous studies have reported the association of off-pump CABG with the reduced requirement of transfusion in patients with left ventricular dysfunction [35]. However, No improvement in neurocognitive outcomes after off-pump versus on-pump coronary revascularization [36].

The off-pump CABG involves less hypercoagulable state and thromboembolic events, thus reducing micro emboli, activation of the coagulation and inflammatory cascades [37]. Yeatman et al. reported that the patients undergoing either off-pump CABG or on-pump CABG for LVD show that off-pump CABG displayed lower requirements for inotropes, less transfusion requirement, and a slightly shorter hospital stay, but at the price of less complete revascularization [38]. Sawada et al. found that coronary revascularization improves long-term survival and a wide range of viability in 274 patients with ischemic left ventricular dysfunction [39]. Jarral et al. found that the preoperative LVEF had adverse effect on long-term survival of patients with LVD and the long-term survival of patients with severe LVD was significantly lower than those with mild to moderate LVD [40]. But Reid et al. demonstrated that the clinical outcome is improved by surgical revascularization can reduce organ dysfunction which also can improve survival [41].

Many preoperative factors were found to be associated with mortality in CABG with LVD including female sex, increasing older age, diabetes, and peripheral vascular disease as predictors [42, 43]. Margo et al. found that the age (>70 years) and female influences on the needs, concerns, and strategies of CABG caregivers. The effect of CABG on all-cause mortality tended to diminish with increasing age through a more significant burden of comorbidities, which in turn lead to a higher risk of postoperative complications and non-cardiovascular deaths [43]. Both short-and long-term cardiac outcomes of odd-pump CABG are not influenced by age at the operation which prevents the potential complications that can occur in patients undergoing CABG with CPB [44]. The surgeon experience also the essential factors for the clinical outcome of CABG which improved by surgical technique, surgeon volume, and hospital volume, changed surgical training [45–48].

### Limitations

There are many limitations should be acknowledged. Firstly, the number of patients, the inclusion and the exclusion criteria, the type of surgery, the indication for CABG, methods for the assessment of LV function and the definition of the severe LVD varied across the studies, and the EF has represented a systolic function which cannot be demonstrated left ventricular dimension and diastolic function. Secondly, the surgeon’s volume index and institutional volume index also were not significantly associated with the clinical outcome because the learning curve of off-pump CABG is longer than on-pump CABG. Finally, the present study remains subject to the inherent caveats of a meta-analysis including publication bias, however, in-depth statistical analysis was performed to account for these limitations. In future, the more RCT studies need to studies the clinical outcome of OPCAB and ONCAB.

## Conclusions

The published evidence on the clinical effect of the use the off-pump CABG for LVD is mainly derived single-center observational studies from the institutions. The key finding is that the use of off-pump CABG is associated with a reduction in mortality and this finding also provide better implications for clinicians and policymakers .

## Supplementary information

**Additional file 1.** Search strategy.

**Additional file 2: Figure S1.** a-1b 30-day mortality: (a) Funnel plot with Egger test results and (b) Leave-one-out analysis.

## Data Availability

Not applicable.

## Abbreviations

LVD: Left ventricular dysfunction
CABG: Coronary artery bypass grafting
CNS: Central nervous system complications
ICU LOS: The length of stay of an intensive care unit
CPB: Cardiopulmonary bypass
MED: Medical therapy
AKI: Acute kidney disease
CKD: Chronic kidney disease
