# Supplementary material for: Short-term outcomes of on- vs off-pump coronary artery bypass grafting in patients with left ventricular dysfunction: a systematic review and meta-analysis
Source: J Cardiothorac Surg. 2020 May 11;15:84. doi: 10.1186/s13019-020-01115-0 (PMC7216614; doi:10.1186/s13019-020-01115-0)
Supplement: Supplementary file 1 — Additional file 1. Search strategy. [file 13019_2020_1115_MOESM1_ESM.pdf]

Search strategy

((((((((((((coronary artery bypass grafting[Title/Abstract]) OR coronary artery bypass surgery[Title/Abstract]) OR CABG[Title/Abstract]) OR on-pump[Title/Abstract]) OR conventional[Title/Abstract]) OR cardiopulmonary bypass[Title/Abstract])) OR “Coronary Artery Bypass”[Mesh])) AND (((off-pump[Title/Abstract]) OR cardioplegia[Title/Abstract]) OR OPCAB[Title/Abstract]))) AND ((((((((short term[Title/Abstract]) OR 30 day[Title/Abstract]) OR thirty day[Title/Abstract]) OR perioperative[Title/Abstract]) OR early[Title/Abstract]) OR operative[Title/Abstract]) OR postoperative[Title/Abstract]) OR in-hospital[Title/Abstract]))) AND ((((“Ventricular Dysfunction, Left”[Mesh]) OR (((poor left ventricle[Title/Abstract]) OR ("ejection fraction" [Title/Abstract] OR "EF"[Title/Abstract]) (ischemic cardiomyopathy[Title/Abstract])))) OR ((((left ventricular[Title/Abstract]) OR LV[Title/Abstract])) AND ((((dyssynchrony[Title/Abstract]) OR dysfunction[Title/Abstract]) OR compromised[Title/Abstract]) OR depressed[Title/Abstract]))))
