# Supplementary figures and images for: Short-term outcomes of on- vs off-pump coronary artery bypass grafting in patients with left ventricular dysfunction: a systematic review and meta-analysis
Source: J Cardiothorac Surg. 2020 May 11;15:84. doi: 10.1186/s13019-020-01115-0 (PMC7216614; doi:10.1186/s13019-020-01115-0)

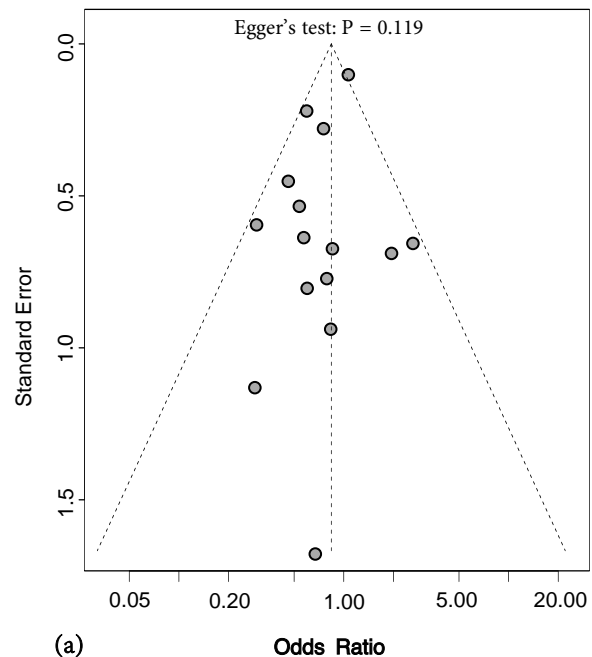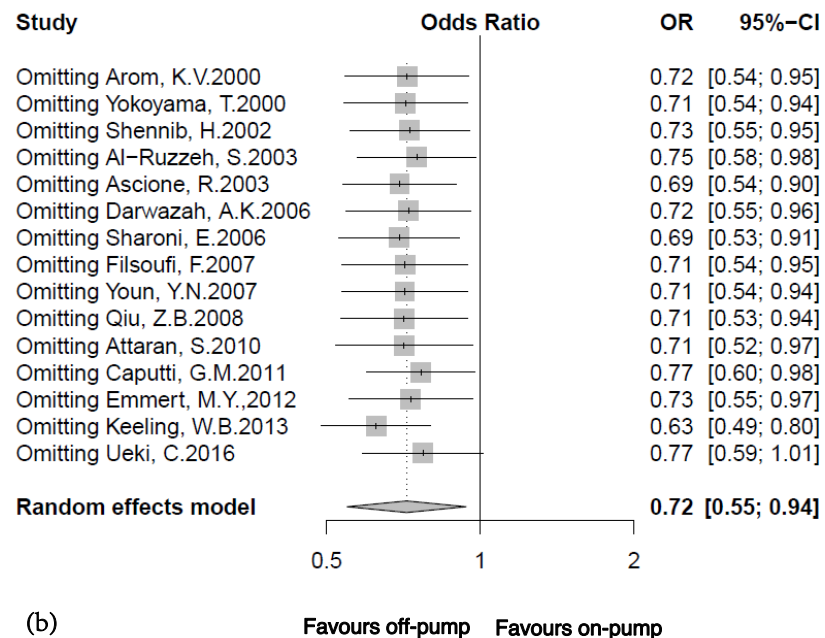

Supplement: Supplementary file 2 — Additional file 2: Figure S1. a-1b 30-day mortality: (a) Funnel plot with Egger test results and (b) Leave-one-out analysis. [file 13019_2020_1115_MOESM2_ESM.pdf]
